# Supplementary material for: Miocene sponge assemblages in the face of the Messinian Salinity Crisis—new data from the Atlanto-Mediterranean seaway
Source: PeerJ. 2023 Nov 16;11:e16277. doi: 10.7717/peerj.16277 (PMC10657567; doi:10.7717/peerj.16277)
Supplement: Supplemental Information 1 [file peerj-11-16277-s001.docx]

| **Taxon** | **Name or description** | **Age and locality** | **Reference and remarks** |
| --- | --- | --- | --- |
| *Plocamione* cf. *dirrhopalina* | Curved dumb-bell-like spicules with sculptured heads | Eocene of Ukraine | Łukowiak et al., 2019 |
|  | Straight dump-bell-like spicules with well-developed ornamented heads and central spinose ring | Cretaceous of Sardinia | Łukowiak et al., 2022a |
|  | Stubby cricorhabds | Jurassic | Reif, 1967; Trammer, 1982; Pisera, 1997 |
|  |  | Cretaceous | Moczydłowska & Paruch-Kulczycka, 1978 |
| *Rhabderemia* sp. | Rhabdostyles (in general) |  | Wiedenmayer, 1994 |
|  | Ornamented rhabdostyles | Miocene of Blake-Bahama Basin | Bukry, 1978 |
|  |  | Eocene of New Zealand | Hinde & Holmes, 1892 |
| *Bubaris subtyla* | Flexuous anisoxeas | Mesozoic | Reif, 1967; Mostler, 1986 |
|  |  | Cenozoic | Łukowiak, 2015; Łukowiak & Pisera, 2017; Łukowiak et al., 2019, 2022b |
| *Monocrepidium vermiculatum* | Vermicular diactines | Cretaceous of N Germany | Schrammen, 1924 |
|  |  | Australia | Łukowiak, 2015; Łukowiak & Pisera, 2017 |
|  |  | Miocene of Slovakia | Łukowiak et al., 2014 |
| *Spiroxya* sp. | Spiral strongyles | Miocene of Blake-Bahama Basin | Bukry, 1978 |
|  | Microspined strongyles (shorter) | Cretaceous of Sardinia | Łukowiak et al., 2022b |
| *Diplastrella* sp. |  | Eocene of New Zealand and Australia | Hinde & Holmes, 1892; Łukowiak, 2015 (but without exact assignments) |
| *Placospongia decorticans* | Selenasters | Mesozoic from all over the world | Hinde, 1893; Schrammen, 1936; Hartman, 1980; Trammer, 1982; Pisera 1997, and Carter, 1871; Gruber & Reitner, 1991; Łukowiak et al., 2022b |
|  |  | Cenozoic from all over the world | Hinde, 1910; Rich, 1958; Bukry, 1978; Rigby & Smith, 1992; Pisera & Hladilová, 2003; Pisera et al., 2006; Frisone et al., 2014; Łukowiak, 2015; Łukowiak et al., 2019, 2022b; |
|  |  |  | For more details see Cárdenas 2020 |
| ?*Hamacantha* (*Hamacantha*) *lundbecki* | Diancistras | Jurassic of Austria | Mostler, 1990 |
|  |  | Cretaceous of N Germany | Schrammen, 1924 |
|  |  | Eocene of New Zealand | Hinde & Holmes, 1892 |
| ?*Hamacantha* (*Vomerula*) *papillata* | See above |  |  |
| *Hamacantha* (*Hamacantha*) *johnsoni* | See above |  |  |
| *Histodermella* cf. *ingolfi* | Similar spicules | Eocene of Australia | Łukowiak, 2015 |
| *Crambe* cf. *tuberosa* | Sphaeroclones | Eocene of New Zealand | Hinde & Holmes,1892 |
| *Discorhabdella tuberosocapitata* | Tylostyles with ornamented heads | Eocene of New Zealand and Ukraine | Hinde & Holmes, 1892 Łukowiak et al., 2019 |
|  | Acanthotylostyles | Miocene, waters between New Zealand and Australia | Locker & Martini, 1986 |
| *Discorhabdella* sp. | Acanthostyles (slenderer) | Eocene of Ukraine | Łukowiak et al., 2019 |
|  | Korynasters | Cretaceous of N Germany | Scharammen, 1924 (assigned as belonging to Monaxonida) |
| Latrunculiidae indet. 1 | Aciculospinorhabds |  | Wiedenmayer, 1994 |
|  | Similar, but not identical rhabds | Eocene of New Zealand | Hinde & Holmes, 1892 |
| Latrunculiidae indet. 2 | Discorhabds (“compressed” in the vertical axis) | Eocene of New Zealand | Hinde & Holmes, 1892 |
| Poecilosclerida indet. | Poecilosclerida spicules |  | Wiedenmayer et al., 1994; Pisera, 2006; |
| *Sceptrella* cf. *biannulata* | Strongyles (greater number of coils with spines) | Eocene of Australia | Łukowiak, 2015 (assigned to *Sceptrintus richardi*) |
| *Mycale* (*Mycale*) cf. *grandis* | Anisochelae | Eocene of New Zealand and Ukraine | Hinde & Holmes, 1892; Łukowiak et al., 2019 |
| *Mycale* (*Rhaphidotheca*) *marshallhalli* | Exotyles | Miocene of N Atlantic | Bukry, 1979 |
|  | Club shaped spicules | Eocene | Łukowiak, 2015; Łukowiak et al., 2019 (but no taxonomic assignment) |
| *Euchelipluma pristina* | Placochelae | Eocene of New Zealand | Hinde & Holmes, 1892 |
|  |  | Miocene of Iceland | Bukry, 1979 |
| *Acarnus* sp. | Cladotylotes | Eocene of New Zealand and Australia | Hinde & Holmes, 1892; Łukowiak, 2015 |
|  |  | Miocene of Blake-Bahama Basin | Bukry, 1978 |
| *Antho* sp. | Acanthostrongyles | Jurassic of Austria | Mostler, 1990 |
|  |  | Miocene of Slovakia | Łukowiak et al., 2014 |
| *Clathria* sp. | Long styles with well pronounced (sometimes ornamented) heads | Eocene of Australia and Ukraine | Łukowiak, 2015; Łukowiak et al., 2019 |
|  |  | Cretaceous of N Germany | Schrammen, 1924 (assigned to Monaxonida) |
| *Tethya* sp. | Oxyasters | Miocene of Slovakia | Łukowiak et al., 2014; Costa et al., 2021 |
|  |  | Eocene of Ukraine | Łukowiak et al., 2019 |
| *Annulastrella* cf. *ornata* | Annulated triods | Miocene of Blake-Bahama Basin | Bukry, 1978 |
|  |  | Mesozoic of Europe | Schrammen, 1924; Reif, 1967; Moczydło-wska & Paruch-Kul-czycka, 1978; Mostler, 1990; Pisera, 1997 |
|  | Similar annulated spicules | Triassic and Cretaceous | Maldonado, 2002 (assigned to *Monilites*) |
| *Alectona* cf. *millari* | Alectona acanthoxeas | Eocene of Australia | Łukowiak, 2015 |
|  |  | Miocene of Slovakia | Łukowiak et al., 2014 |
|  | Alectonid-like spicules | Miocene of Bahamas | Burky, 1978 |
| *Thrombus abyssi* | Trichotriaenes | Cretaceous of N Germany | Schrammen, 1924 |
|  | Acanthotrichotriaenes | Eocene of New Zealand | Hinde & Holmes, 1892 |
|  |  | Miocene of Slovakia | Łukowiak et al., 2014 |
| Tetillidae indet. | Anatriaenes |  | See Wiedenmayer, 1994 |
| Astrophorina indet. | Spicules assigned as Astrophorina |  | See Wiedenmayer, 1994 and Cárdenas, 2020 |
| “Lithistida” indet. | “Lithistid” spicules | At least since Cambrian | For more details see Pisera, 2006 |
| Lychniscosida indet. | Lychniscosan skeletons | At least since Jurassic | For more details see Pisera, 2006 |
| *Nodastrella* cf. *nodastrella* | stauractines | Eocene of Australia and Ukraine | Łukowiak, 2015; Łukowiak et al., 2019 |
|  | spicules similar to discasters | Eocene of New Zealand | Hinde & Holmes, 1892 |
| Rossellidae indet. | pentactine | Eocene of Australia | Łukowiak, 2015 (assigned to *Rossella*) |
| Sceptrulophora indet. | dictyonal skeletons | At least since Devonian | Rigby et al., 2001 |
| *Tretopleura* sp. | very similar skeleton fragments with a claw-like projections | Oligoene and Miocene of Bahamas | Palmer, 1988 (but assigned to radio-larians; pl. 3, figs 2, 3) |
| Hexactinellida indet. | Pinules | Cretaceous of N Germany | Schrammen, 1924 |

References

Bukry D. 1978. Cenozoic coccolith, silicoflagellate, and diatom stratigraphy, Deep Sea Drilling Project Leg 44. In: Benson WE, Sheridan RE. et al. eds. *Initial Reports of the Deep Sea Drilling Project*, 44, pp. 807–864. U.S. Government Printing Office, Washington http://dx.doi.org/10.2973/dsdp.proc.44.137.1978.

Bukry D. 1978. Coccolith and silicoflagellate stratigraphy, northern mid-Atlantic Ridge and Reykjanes Ridge, Deep Sea Drilling Project Leg 49. Initial reports of the Deep Sea Drilling Project 49: 551–581 <http://dx.doi.org/10.2973/dsdp.proc.49.118.1979>.

Cárdenas P. 2020. Surface microornamentation of demosponge sterraster spicules, phylogenetic and paleontological implications. *Frontiers in Marine Science* 7:613610.

Carter HJ. 1871. On fossil sponge-spicules of the Greensand compared with those of existing species. *Annals and Magazine of Natural History*, Series 4, 7(38):112–141.

Costa G, Bavestrello G, Cattaneo-Vietti R, Dela Pierre F, Lozar F, Natalicchio M, Violanti D, Pansini M, Rosso A, Bertolino M. 2021. Palaeoenvironmental significance of sponge spicules in pre-Messinian crisis sediments, Northern Italy. *Facies* 67:9. <https://doi.org/10.1007/s10347-020-00619-4>.

Frisone V, Pisera A, Hajdu E, Preto N, Zorzi F, Zorzin R. 2014. Isolated spicules of Demospongiae from Mt. Duello (Eocene, Lesssini Mts, northern Italy): preservation, taxonomy and depositional environment. *Facies* 60:883–904.

Gruber G, Reitner J. 1991. Isolierte Mikro- und Megaskleren von Porifera aus dem Untercampan von Hover (Norddeutschland) und Bemerkungen zur Phylogenie der Geodiidae (Demospongiae). *Berliner Geowissenschaftliche Abhandlungen* 134:107–117.

Hartman WD. 1980. Systematics of the Porifera. pp. 24–51. In: Hartman WD, Wendt JW., Gray JE, eds. Notes on the Arrangement of Sponges, with the Descriptions of some New Genera. Proceedings of the Zoological Society of London, 1867, pp. 492–558.

Hinde GJ. 1893. A monograph of the British fossil sponges. Part III. Sponges of the Jurassic strata. *Palaeontolical Society Monograph* 1:189–254 http://dx.doi.org/10.5962/bh

Hinde GJ. 1910. On the fossil sponge spicules in a rock from deep Lead at Princess Royal Township, Norseman District, Western Australia. *Bulletin of Geological Survey of Western Australia* 36:7–24.

Hinde GJ, Holmes WM. 1892. On the sponge remains in the Lower Tertiary Strata near Oamaru, Otago, New Zealand. *Journal of the Linnean Society of London* 24:177–262. <http://dx.doi.org/10.1111/j.1096-3642.1892.tb02480.x>

Locker S, Martini E. 1986. Ebridians and actiniscidians from the Southwest Pacific. In: Kennett JP, von der Borch CC, et al., eds. Initial Reports of the Deep Sea Drilling Project, Washington (U.S. Govt. Printing Office), 90:939–951.

Łukowiak M. 2015. Late Eocene siliceous sponge fauna of southern Australia: reconstruction based on loose spicules record. *Zootaxa* 3917(1):1–65.

Łukowiak M, Dieni I, Dumitrica P, Massari F. 2022b. Late Valanginian sponge spicules from north-eastern Sardinia (Italy). *Cretaceous Research* 135:105205.

Łukowiak M, Soest R van, Klautau M, Pérez T, Pisera A, Tabachnick K. 2022a. The terminology of sponge spicules. *Journal of Morphology* 283(12):1517–1545 <https://doi.org/10.1002/jmor.21520>.

Łukowiak M, Pisera A. 2017. Bodily preserved Eocene non-lithistid demosponge fauna from southern Australia: taxonomy and affinities. *Journal of Systematic* *Palaeontology* 15(6):473–497.

Łukowiak M, Pisera A, Schlögl J. 2014. Bathyal sponges from the late Early Miocene of the Vienna Basin (central Paratethys, Slovakia). *Paläontologische Zeitschrift* 88:263–277.

Łukowiak M, Pisera A, Stefanska T. 2019. Uncovering the hidden diversity of Paleogene sponge fauna of the East European Platform through reassessment of the record of isolated spicules. *Acta Palaeontologica Polonica* 64(4):871e895 <https://doi.org/10.4202/app.00612.2019>.

Moczydłowska M, Paruch-Kulczycka J. 1978. Analiza spikul gąbek krzemionkowych z oksfordu Wrzosowej i Zawodzia oraz z kampanu Bonarki. *Kwartalnik Geologiczny* 22:83–103.

Mostler H. 1986 (1985). Beitrag zur stratigraphischen Verbreitung und phylogenetischen Stellung der Amphidiscophora und Hexasterophora (Hexactinellida, Porifera*). Mitteilungen Der Östereichischen Geologischen Gesellschaft* 78:319–359.

Mostler H. 1990. Mikroskleren von Demospongien (Porifera) aus dem basalen Jura der Nördlichen Kalkalpen. *Geologisch- Paläontologische Mitteilungen Insbruck* 17:119–142.

Palmer A. 1988. Paleoenvironmental significance of siliceous sponge spicules from sites 627 and 628, little Bahama Bank, Ocean Drilling Program leg 1011. In: Austin, JA, Jr, Schlager W, et al., eds. *Proceedings of the Ocean Drilling Program, Scientific Results* 101:10.

Pisera A. 1997. Upper Jurassic siliceous sponges from the Swabian Alb: taxonomy and paleoecology. *Palaeontologia Polonica* 57:1–216.

Reif W-E. 1967. Schwammspicula aus dem Weissen Jura Zeta von Natheim (Schwabische Alb). *Palaeontographica* 127:85–102.

Rich CC. 1958. Occurrence of sterrasters of the Geodiidae (Demospongea, Choristida) in late Cenozoic strata of western Wellington Province, New Zealand. *New Zealand Journal of Geology and Geophysics* 1:641–646 <http://dx.doi.org/10.1080/00288306.1958.10423172>

Rigby JK, Pisera A, Wrzolek T, Racki G. 2001. Upper Devonian sponges from the holy cross mountains, central Poland. *Palaeontology* 44:447–488.

Rigby JK, Smith CC. 1992. Microscleres of a Paleocene *Geodia* from western Alabama. *Journal of Paleontology* 66(3):406–413.

Schrammen A. 1924. Die Kieselspongien der oberen Kreide von Nordwestdeutschland. III und letzter Teil. Mit Beiträgen zur Stammesgeschichte. *Monographien zur Geologie und Paläontologie* 1:1–159.

Schrammen A. 1936. Die Kieselspongien des oberen Jura von Suddeutschland. A. Vorwort und Allgemeiner Teil. *Palaeontographica* 84:49–194.

Trammer J. 1982. Lower to Middle Oxfordian sponges of the Polish Jura. *Acta Geologica Polonica* 32:1–39.

Wiedenmayer F. 1994. Contribution of the knowledge of post-Paleozoic neritic and archibenthal sponges (Porifera). *Schweizerische Paläontologische Mitteilungen* 116:1–147.
