# Supplementary material for: Miocene sponge assemblages in the face of the Messinian Salinity Crisis—new data from the Atlanto-Mediterranean seaway
Source: PeerJ. 2023 Nov 16;11:e16277. doi: 10.7717/peerj.16277 (PMC10657567; doi:10.7717/peerj.16277)
Supplement: Supplemental Information 2 — AS—Alboran Sea, WM—Western Mediterranean, AZ—Azores, NA—North Atlantic, RS—Red Sea, SEAS—South European Atlantic Shelf. [file peerj-11-16277-s002.docx]

| **Species** | **Modern distribution** | **Depth [m]** |
| --- | --- | --- |
| *Plocamione dirrhopalina* | WM | 1200 |
| *Bubaris subtyla* | SEAS | 120–150 |
| *Monocrepidium vermiculatum* | WM | 121–600 |
| *Placospongia decorticans* | SEAS | Shallow |
| *Hamacantha* (*H.*) *lundbecki* | SEAS | 780 |
| *Hamacantha* (*V.*) *papillata* | SEAS | 185–1600 |
| *Hamacantha* (*H.*) *johnsoni* | SEAS | 170–924 |
| *Histodermella ingolfi* | NA | 700–1400 |
| *Crambe* *tuberosa* | AS | 70–120 |
| *Discorhabdella tuberosocapitata* | AZ | 534–604 |
| *Sceptrella biannulata* | AZ | 30 |
| *Mycale* (*Mycale*) *grandis* | RS | Very shallow |
| *Mycale* (*R.*) *marshallhalli* | SEAS | 75–900 |
| *Euchelipluma pristina* | NA | 91 |
| *Annulastrella ornata* | AS | 123–147 |
| *Alectona millari* | SEAS | 400 |
| *Thrombus abyssi* | SEAS | 1378 |
| *Nodastrella nodastrella* | AZ | 760–1400 |
| *Spiroxya spiralis* | AZ | 12–30 |
